# Analytical Modeling Tool for Design of Hydrocarbon Sensitive Optical Fibers

**DOI:** 10.3390/s17102227

**Published:** 2017-09-28

**Authors:** Khalil Al Handawi, Nader Vahdati, Oleg Shiryayev, Lydia Lawand

**Affiliations:** Department of Mechanical Engineering, Khalifa University of Science and Technology, Petroleum Institute, P.O. Box 2533 Abu Dhabi, UAE; khbalhandawi@pi.ac.ae (K.A.H.); oshiryayev@pi.ac.ae (O.S.); lyslawand@pi.ac.ae (L.L.)

**Keywords:** distributed sensing, optical time domain reflectometry, polymer clad silica fibers, corrosion monitoring, oil pipelines, pipeline integrity management

## Abstract

Pipelines are the main transportation means for oil and gas products across large distances. Due to the severe conditions they operate in, they are regularly inspected using conventional Pipeline Inspection Gages (PIGs) for corrosion damage. The motivation for researching a real-time distributed monitoring solution arose to mitigate costs and provide a proactive indication of potential failures. Fiber optic sensors with polymer claddings provide a means of detecting contact with hydrocarbons. By coating the fibers with a layer of metal similar in composition to that of the parent pipeline, corrosion of this coating may be detected when the polymer cladding underneath is exposed to the surrounding hydrocarbons contained within the pipeline. A Refractive Index (RI) change occurs in the polymer cladding causing a loss in intensity of a traveling light pulse due to a reduction in the fiber’s modal capacity. Intensity losses may be detected using Optical Time Domain Reflectometry (OTDR) while pinpointing the spatial location of the contact via time delay calculations of the back-scattered pulses. This work presents a theoretical model for the above sensing solution to provide a design tool for the fiber optic cable in the context of hydrocarbon sensing following corrosion of an external metal coating. Results are verified against the experimental data published in the literature.

## 1. Introduction

Oil transfer pipelines are used as a means for the transportation of crude oil mixtures to designated processing facilities. Such pipelines are extensively used in the UAE and span large distances [1] while being subject to adverse weather conditions. These conditions are characterized by high temperatures and high levels of ambient humidity during the summer season. As a result, these structures are constantly attacked by the various mechanisms of corrosion both internally and externally, providing motivation to monitor these large structures for corrosion damage.

Conventionally, several standardized monitoring tools are available to pipeline operators for the purpose of inspection [2]. Most internal monitoring tools involve intruding the pipeline. A Pipeline Inspection Gage (PIG) is routinely used for cleaning pipelines. Modern PIGs are instrumented for wall thickness and surface defect detection. The sensors used employ Magnetic Flux Leakage (MFL) as a tool for inferring wall thickness along the pipelines’ axial direction [3]. “Smart” PIGs conduct their inspection runs on off-line pipelines, which disrupts operation. Additionally, visual inspection probes may be administered into the pipeline once it is off-line. Closed Circuit TV (CCTV) technology is used to map the internal surface and collect data about its condition. Similarly, the pipeline must be off-line so that the probe may be administered into it for inspection [4].

In order to mitigate the need for disturbing pipeline operation, disposable corrosion coupons have been employed so that they may continuously monitor the corrosion rate as opposed to the periodic inspection intervals offered by the previous tools. Corrosion coupons are inserted into the pipeline and retrieved using a retrieval valve and tool. Once in position, the corrosion current generated by the oxidation of iron may be used to continuously infer the corrosion rate on the internal side of the pipeline [5]. Although this technique offers continuous monitoring of the pipeline conditions without disturbing operation, the required retrieval valves involve extensive retrofitting costs for existing pipelines, making them economically impractical as a monitoring solution. In addition to cost, corrosion coupons have other disadvantages, and they are:

- The corrosion coupons may not be experiencing the same flow regime as the pipe surface, thus indicating different corrosion rates than the pipelines.
- Build-up of scale, asphaltene and wax on the corrosion coupons can impact the true corrosion rate accuracy, particularly if the buildup occurs on the coupons, but not on the interior surface of the pipeline.
- Removal of the corrosion coupons from the pipelines for inspection can lead to deaths due to the release of toxic gases (such as H${}_{2}$S, etc.) from pipelines.

Due to the above shortcomings of conventional inspection tools and due to the volatile operating environment within pipelines, a sensing element and communication medium that are intrinsically safe for live pipelines have been targeted as a likely solution. Optical Fiber Sensors (OFS) have been used for Structural Health Monitoring (SHM) in the past and would likely overcome the above shortcomings. Optical fibers are the most natural and logical sensors to be used in the oil and gas industry since data are transmitted through the fiber using pulses of light, the data transfer is extremely fast and light cannot cause sparks and fires. Optical fibers are also immune from electromagnetic interference.

OFS have been categorized into long gage, point, quasi distributed and distributed sensors [6]. Long Period Grating (LPG) sensors are used in civil structures to monitor displacement of a taught sensing fiber through phase modulation against a reference fiber using interferometry. Fiber Bragg Grating (FBG) sensors are used to monitor local strains and temperatures in civil structures using wavelength modulation of a broadband light pulse in relation to distortion of the grating spacing. Other point sensors such as micro and macro bend sensors have also been reported [6,7].

Some of the above-mentioned sensor topologies have found applications specifically in corrosion sensing. Optical fibers have been used as pH sensors via immobilized dyes that have an absorption spectrum sensitive to hydrogen anions. Spectroscopic methods may be used to detect the hydrogen ion concentration and hence the pH level that is strongly related to corrosion. Other approaches utilize LPGs whose cladding is spectrally sensitive to sodium chloride concentrations. Stripped cladding sensors utilizing thermally-deposited metal coatings may be used to detect its removal as transmitted power is restored to previous levels before applying the metal cladding [8,9,10]. Point reflectivity probes may be used to scan the surfaces of pipes and walls to detect surface damage or bacterial colonies [6,11].

Finally, truly distributed fiber optic sensing networks have been realized using backscatter reflectometry in which the time delay between firing a laser pulse and receiving the backscattered pulse along various locations on the fiber is resolved to provide spatial information. The spectral profile of the backscattered light pulse may be used to infer temperature and strain effects by virtue of Rayleigh, Brillouin or Raman scattering [6,12].

Distributed fiber optic sensors have found several applications in pipeline leakage detection. One technique performs distributed temperature measurements via Raman spectroscopy in order to detect leakage from a nearby pipeline transporting fluids at a higher temperature relative to the surroundings [13]. Another technique performs distributed acoustic measurements via the Refractive Index (RI) modulation due to strains. The RI change and its location can be inferred using distributed Sagnac interferometry by interpreting the generated phase signal [14].

Further investigation into distributed fiber optic sensors yielded an approach used for hydrocarbon leakage detection via Optical Time Domain Reflectometry (OTDR). A polymer-clad fiber is sensitive to the hydrocarbons due to the swelling of the cladding and the changes in its RI as a result of hydrocarbon absorption. OTDR is used to measure the loss in light intensity due to chemical perturbations in a polymer-clad fiber that alter its RI [15].

Due to the abundance of hydrocarbons in oil pipelines and the need for corrosivity detection in the internal environment of the pipeline, a hybrid method combining metal-coated fibers with the polymer-clad fibers to detect excessive corrosivity levels is theoretically investigated. The technique employs a high power laser diode that fires a laser pulse through the fiber under inspection [16]. OTDR is used to resolve the location of metal loss from the coating following hydrocarbon absorption into the now exposed cladding. A change in the attenuation coefficient characterizes the losses of a traveling light pulse.

A theoretical investigation of the above phenomenon was explored by the authors in [17]. The results presented are limited in terms of validation and do not provide sensing predictions in the context of internal corrosion sensing of pipelines by virtue of hydrocarbon contact. This work extends [17] by providing a more thorough theoretical tool that is verified against experimental results from the literature and is used to provide a calibration curve that is useful for designing the proposed sensing solution for maximum sensitivity in crude oil pipelines.

## 2. Methodology and Sensor Operation Principle

### 2.1. Sensor Deployment

It is envisioned that the optical fiber corrosion sensor, proposed in this paper, will be deployed in the oil pipelines either through the PIG launcher or retrieval stations or through valves. The work in [18] shows a proven method to install fiber optic cable inside gas pipelines using existing valves. The fixtures, proposed in [18], have been designed to provide gas-tight seals around the cable entry and exit points. Just like [18], it is envisioned that our proposed optical fiber corrosion sensor will lay on the bottom of the pipeline very near the surface of the pipeline; therefore, seeing the same flow regime as the pipe surface; thus, experiencing similar corrosion rates as the oil pipelines. If sediments such as scale, wax, or asphaltene form in the interior surface of the pipe, since the optical fiber is also very near the pipe surface, it will experience the same phenomenon.

The transduction principle of the proposed sensor relies on a break in the metal coat due to excessive corrosion. Upon exposure of the polymer cladding underneath, hydrocarbons in the surrounding medium diffuse into the polymer, causing its RI to change. This problem will be modeled by utilizing the established theoretical models for light intensity distribution and a specially-developed numerical model for arbitrary RI profiles as explained in the following sections.

From a practical point of view, it is worth noting that the diffusion of hydrocarbons into the cladding is indeed reversible and does not affect the integrity of the fiber. The work in [15] (Section 3.3) makes use of this phenomena to regenerate the same fiber after testing by evaporating the volatile diffused hydrocarbons in air or acetone. We must note that the model presented in this paper does not account for the effects of swelling.

Another practical aspect of the proposed methodology could be the temperature sensitivity of the cladding RI to the high temperatures encountered within oil transfer lines. A study by [19] on photonic Polydimethylsiloxane (PDMS) crystals shows a very small thermo-optic effect (variation of RI with temperature). PDMS will be the material of choice for the cladding of the fiber for reasons explained in subsequent sections. RI changes with temperature were in the order of 10${}^{-5}$, which are unlikely to have an appreciable effect on the attenuation of the signal. The work in [20] mentions in an experimental study and characterization that RI of light crude mixture varies from 1.4530 at 20 ${}^{\circ }$C to 1.4379 at 60 ${}^{\circ }$C. In large transfer lines, the oil will be closer to the surrounding temperature of 40–50 ${}^{\circ }$C, which is within the range of RI values included in subsequent calculations within this paper.

From a fabrication point of view, uniform deposition of metal, similar to the pipeline, can be achieved on optical fibers using the sputtering technique (physical vapor deposition). Many fiber optics companies already provide optical fibers with polyamide, gold, silver, copper, copper nickel alloy, aluminum, platinum and palladium coatings. It should be possible to coat the optical fibers with steel from the parent pipeline (API 5L X-grade material). Coating thicknesses, currently used by the fiber optics companies, range from 15 μm for single-mode fibers to 60 μm for large-core multi-mode fibers. We believe that thicker coatings suitable for this application are feasible because metallic deposition technology is currently widely used for manufacturing of automotive trim, optical components and even bathroom fixtures.

**Figure 1:**
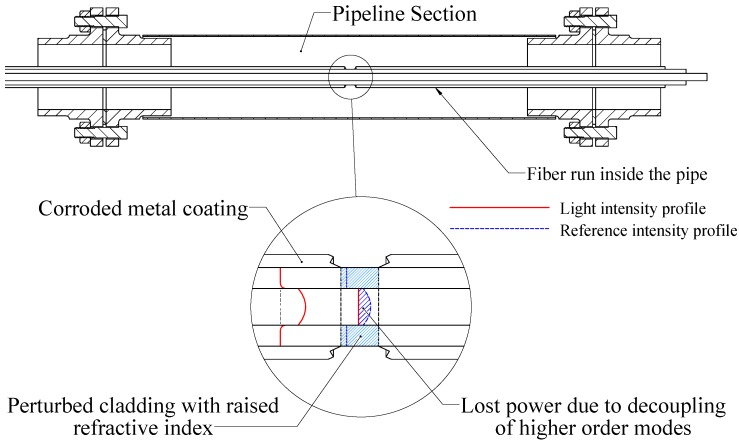
Operation principle of the distributed fiber optic corrosion sensor when exposed to hydrocarbons showing the resulting light intensity distributions.

A schematic of the perturbed fiber is shown in Figure 1.

### 2.2. Mathematical Modeling of Light Intensity Distributions

The paper will introduce several mathematical models for the diffusion of hydrocarbons into the polymer cladding. A model for the calculation of the resulting modal capacity and light intensity profiles due to the diffusion of the hydrocarbons into the cladding is then developed. Finally, the resulting loss in intensity from a reference fiber configuration (before hydrocarbon intrusion) is computed in each case and compared to experimental measurements found in the literature.

A mathematical model is sought to calculate the light intensity distribution for arbitrary RI profiles in order to compare the resulting power to that carried by a reference case and devise an attenuation coefficient representative of the perturbation. Following the derivations presented in the literature [21,22,23], the following derived models build on the established governing equations to define the ordinary differential equations that describe the light intensity profile while holding the RI parameter, an arbitrary function of radius. Analytical results of the governing equations (Maxwell’s equations) in the literature describe intensity distributions for fixed and quadratic core refractive index profiles. However, no general purpose method exists for solving these differential equations in the case of arbitrary RI profiles (especially in the cladding). The resulting RI profiles due to hydrocarbon concentration gradients in the cladding could result in such arbitrary RI profiles. Hence, a numerical tool suited for this case will be developed in Section 2.2.2.

#### 2.2.1. Derivation of General Light Intensity Field Distributions in a Circular Fiber Optic Waveguide

To begin with, the assumed solutions of the light intensity distribution given by Equations (1) and (2) for the core and cladding regions respectively are substituted into the governing equations (please see the Supplementary Information for a review of the derivations of the wave equation from Maxwell’s equations).

(1) $$\begin{array}{c} e_{t}\left(r,\phi \right)=e_{co}\left(r\right)\cos l\phi \hat{x}\;for\;r\leq a_{core}\;, \end{array}$$

(2) $$\begin{array}{c} e_{t}\left(r,\phi \right)=e_{cl}\left(r\right)\cos l\phi \hat{x}\;for\;r>a_{core}\;. \end{array}$$

$e_{t}$ represents the intensity distribution to be solved for as a function of the radial and azimuthal coordinates used in cylindrical coordinates to define a circular fiber; $e_{co}$ and $e_{cl}$ represent the radial intensity distributions for the core and cladding respectively and are to be solved for. Here, *l* is the azimuthal mode number, which can be any non-negative integer; 0,1,2.….

The Helmholtz equation, which is known as the wave equation in cylindrical coordinates, is derived from Maxwell’s equations. In terms of the defined field intensity distribution, the wave equation is given as follows:

(3) $$\nabla^{2}\left[e_{t}\left(x,\;y\right)\right]e^{-j\beta z}+\mu_{0}\varepsilon_{0}\omega^{2}{n\left(r\right)}^{2}\;\left[e_{t}\left(x,\;y\right)\right]e^{-j\beta z}=0\;,$$

where $\mu_{0}$ is the magnetic permittivity of free space, $\varepsilon_{0}$ is the electrical permittivity of free space, ω is the angular velocity of the light wave, which is derived from the wavelength, β is the propagation constant of the fiber, which is a parameter to be derived from the boundary conditions of the problem, and $n\left(r\right)$ is the RI profile function.

Substituting Equation (1) into Equation (3) yields the first differential equation in the core region:

(4) $$\frac{1}{r}\frac{\partial }{\partial r}\left(r\frac{\partial e_{co}\left(r\right)}{\partial r}\right)+\left(k^{2}n{\left(r\right)}^{2}-\beta^{2}-\frac{l^{2}}{r^{2}}\right)e_{co}\left(r\right)=0\;,$$

where $k^{2}=\mu_{0}\varepsilon_{0}\omega^{2}$. Similarly, substituting Equation (2) into Equation (3) yields the differential equation in the cladding region:

(5) $$\frac{1}{r}\frac{\partial }{\partial r}\left(r\frac{\partial e_{cl}\left(r\right)}{\partial r}\right)+\left(k^{2}n{\left(r\right)}^{2}-\beta^{2}-\frac{l^{2}}{r^{2}}\right)e_{cl}\left(r\right)=0\;.$$

The term $k^{2}n^{2}-\beta^{2}$ is defined as follows for the core region [21]:

(6) $$k^{2}{n_{co}}^{2}-\beta^{2}=\frac{V^{2}}{{a_{core}}^{2}}\left(1-b\right)\;,$$

where *V* is the modal capacity of the fiber defined as $V=ka_{core}\sqrt{{n_{co}}^{2}-{n_{cl}}^{2}}$, $a_{core}$ is the fiber core radius and *b* is the generalized guide index, which is computed from boundary conditions. Equation (6) is used to eliminate β from Equation (4):

(7) $$\frac{1}{r}\frac{\partial }{\partial r}\left(r\frac{\partial e_{co}\left(r\right)}{\partial r}\right)+\left(k^{2}\left(n{\left(r\right)}^{2}-{n_{co}}^{2}\right)+\frac{V^{2}}{{a_{core}}^{2}}\left(1-b\right)-\frac{l^{2}}{r^{2}}\right)e_{co}\left(r\right)=0\;,$$

where $n\left(r\right)$ denotes the arbitrary profile of the RI in the core region of the fiber and $n_{co}$ denotes the nominal RI of the core at the central axis of the fiber.

A similar manipulation is performed on the cladding differential equation knowing that the term $k^{2}n^{2}-\beta^{2}$ is defined as follows for the cladding region [21]:

(8) $$k^{2}{n_{cl}}^{2}-\beta^{2}=-\frac{V^{2}}{{a_{core}}^{2}}b\;.$$

Similarly, Equation (5) is left in terms of $n\left(r\right)$ to accommodate an arbitrary cladding RI, and Equation (8) is used to eliminate the propagation constant β:

(9) $$\frac{1}{r}\frac{\partial }{\partial r}\left(r\frac{\partial e_{cl}\left(r\right)}{\partial r}\right)+\left(k^{2}\left(n{\left(r\right)}^{2}-{n_{cl}}^{2}\right)-\frac{V^{2}}{{a_{core}}^{2}}b-\frac{l^{2}}{r^{2}}\right)e_{cl}\left(r\right)=0\;,$$

where $n\left(r\right)$ denotes the arbitrary profile of the RI in the cladding region of the fiber and $n_{cl}$ denotes the nominal RI of the cladding.

Equations (7) and (9) represent the general differential equations governing the solution for the radial tangential field, which defines the intensity distribution for any particular mode.

#### 2.2.2. Derivation of State Equations to Be Solved Using Numerical Computation

The development of a numerical model for the arbitrary-index fiber involves solving the governing differential equations for the light intensity. The governing equations after expanding Equations (7) and (9) are given by:

(10) $$\begin{array}{cc} \frac{d^{2}e_{co}\left(r\right)}{dr^{2}}+\frac{1}{r}\frac{de_{co}\left(r\right)}{dr}+\left(k^{2}\left(n{\left(r\right)}^{2}-{n_{co}}^{2}\right)+\frac{V^{2}}{{a_{core}}^{2}}\left(1-b\right)-\frac{l^{2}}{r^{2}}\right)e_{co}\left(r\right)=0\; & r\leq a_{core}\\ \frac{d^{2}e_{cl}\left(r\right)}{dr^{2}}+\frac{1}{r}\frac{de_{cl}\left(r\right)}{dr}+\left(k^{2}\left(n{\left(r\right)}^{2}-{n_{cl}}^{2}\right)-\frac{V^{2}}{{a_{core}}^{2}}b-\frac{l^{2}}{r^{2}}\right)e_{cl}\left(r\right)=0\; & r>a_{core}\;. \end{array}$$

However, since the equations are divided on the interval $0\;\leq r\leq a_{core}$ and $r>a_{core}$, each equation needs a change of variables such that both equations share the same parametric variable that ranges from 0–1.

This is done by first changing the first equation in the core region using the following change of variables to ψ:

$$r=a_{core}\times \psi ,\frac{dr}{d\psi }=a_{core}.$$

The equation in the cladding region is given by the following change of variables:

$$r=\psi \left(b_{clad}-a_{core}\right)+a_{core},\frac{dr}{d\psi }=b_{clad}-a_{core}.$$

The state variables for the above equations are:

(11) $$\vec{q}=\left[\begin{array}{c} e_{co}\\ \frac{de_{co}}{dr}\\ e_{cl}\\ \frac{de_{cl}}{dr} \end{array}\right]=\left[\begin{array}{c} e_{co}\\ \frac{1}{a_{core}}\frac{de_{co}}{d\psi }\\ e_{cl}\\ \frac{1}{b_{clad}-a_{core}}\frac{de_{cl}}{d\psi } \end{array}\right]\;,$$

While the derivative $\frac{d\vec{q}}{d\psi }$ denoted as $\nabla \vec{q}$ is:

$$\nabla \vec{q}=\left[\begin{array}{c} \frac{de_{co}}{d\psi }\\ \frac{1}{a_{core}}\frac{d^{2}e_{co}}{d\psi^{2}}\\ \frac{de_{cl}}{d\psi }\\ \frac{1}{b_{clad}-a_{core}}\frac{d^{2}e_{cl}}{d\psi^{2}} \end{array}\right].$$

From Equation (10) and the above transformations, we have:

$$\nabla \vec{q}=\left[\begin{array}{c} \frac{de_{co}}{d\psi }\\ -\frac{1}{a_{core}\psi }\frac{de_{co}}{d\psi }-\left(k^{2}\left(n{\left(r\right)}^{2}-{n_{co}}^{2}\right)+\frac{V^{2}}{{a_{core}}^{2}}\left(1-b\right)-\frac{l^{2}}{{a_{core}}^{2}\psi^{2}}\right)a_{core}e_{co}\\ \frac{de_{cl}}{d\psi }\\ -\frac{1}{\Psi }\frac{de_{cl}}{d\psi }+\left(k^{2}\left(n{\left(r\right)}^{2}-{n_{cl}}^{2}\right)-\frac{V^{2}}{{a_{core}}^{2}}b-\frac{l^{2}}{\Psi^{2}}\right)\left(b_{clad}-a_{core}\right)e_{cl} \end{array}\right]\;,$$

where $\Psi =\psi \left(b_{clad}-a_{core}\right)+a_{core}$ and 0≤ψ≤1. The above equation may be written in the final form in terms of the elements of the vector $\vec{q}$, Equation (11) as:

(12) $$\nabla \vec{q}=\left[\begin{array}{c} a_{core}\vec{q}\left[2\right]\\ -\frac{1}{\psi }\vec{q}\left[2\right]-\left(k^{2}\left(n{\left(r\right)}^{2}-{n_{co}}^{2}\right)+\frac{V^{2}}{{a_{core}}^{2}}\left(1-b\right)-\frac{l^{2}}{{a_{core}}^{2}\psi^{2}}\right)a_{core}\vec{q}\left[1\right]\\ \left(b_{clad}-a_{core}\right)\vec{q}\left[4\right]\\ -\frac{\left(b_{clad}-a_{core}\right)}{\Psi }\vec{q}\left[4\right]+\left(k^{2}\left(n{\left(r\right)}^{2}-{n_{cl}}^{2}\right)-\frac{V^{2}}{{a_{core}}^{2}}b-\frac{l^{2}}{\Psi^{2}}\right)\left(b_{clad}-a_{core}\right)\vec{q}\left[3\right] \end{array}\right]\;.$$

However, the use of a numerical solver would prove difficult in this situation since there are singular terms in the first two elements of $\nabla \vec{q}$; $-{}^{1}/{}_{\psi }\;\vec{q}\left[2\right]$ and ${}^{l^{2}}/{}_{a^{2}\psi^{2}}$ when ψ→0. This problem is circumnavigated by approximating the value of the function $e_{co}$ and $\frac{de_{co}}{d\psi }$ near the singular coordinate (zero) by using a Taylor series approximation for the function and its derivative at a point *d* near the origin as follows:

$$f\left(x-a\right)=f\left(a\right)+\frac{f^{\prime }\left(a\right)}{1!}\left(x-a\right)+\frac{f^{\prime \prime }\left(a\right)}{2!}{\left(x-a\right)}^{2}+\ldots$$

$$e_{co}\left(d\right)=e_{co}\left(0\right)+{e_{co}}^{{}^{\prime }}\left(0\right)d+\frac{{e_{co}}^{{}^{\prime \prime }}\left(0\right)}{2}d^{2}+\ldots$$

$${e_{co}}^{{}^{\prime }}\left(d\right)={e_{co}}^{{}^{\prime \prime }}\left(0\right)d+\ldots ,$$

where, ${e_{co}}^{{}^{\prime }}=\frac{de_{co}}{d\psi }$ and ${e_{co}}^{{}^{\prime \prime }}=\frac{d^{2}e_{co}}{d\psi^{2}}$. ${e_{co}}^{{}^{\prime }}\left(0\right)=0$ due to the symmetric boundary condition at the center of the fiber core, and ${e_{co}}^{{}^{\prime \prime }}$ is obtained from Equation (10) with the coordinates transformed to ψ as follows:

$$\frac{1}{{a_{core}}^{2}}\left({e_{co}}^{{}^{\prime \prime }}+\frac{1}{\psi }{e_{co}}^{{}^{\prime }}\right)=-\left(k^{2}\left(n{\left(a_{core}\psi \right)}^{2}-{n_{co}}^{2}\right)+\frac{V^{2}}{{a_{core}}^{2}}\left(1-b\right)-\frac{l^{2}}{{a_{core}}^{2}\psi^{2}}\right)e_{co}$$

As ψ→0, $\frac{{e_{co}}^{{}^{\prime }}\left(\psi \right)}{\psi }\to {e_{co}}^{{}^{\prime \prime }}\left(0\right)$, hence:

$$\frac{2}{{a_{core}}^{2}}{e_{co}}^{{}^{\prime \prime }}\left(0\right)\approx -\left(k^{2}\left(n{\left(a_{core}\psi \right)}^{2}-{n_{co}}^{2}\right)+\frac{V^{2}}{{a_{core}}^{2}}\left(1-b\right)-\frac{l^{2}}{{a_{core}}^{2}\psi^{2}}\right)e_{co}\left(0\right)$$

$${e_{co}}^{{}^{\prime \prime }}\left(0\right)\approx -\left(k^{2}\left(n{\left(a_{core}\psi \right)}^{2}-{n_{co}}^{2}\right)+\frac{V^{2}}{{a_{core}}^{2}}\left(1-b\right)-\frac{l^{2}}{{a_{core}}^{2}\psi^{2}}\right)\frac{{a_{core}}^{2}}{2}e_{co}\left(0\right)$$

to yield the boundary conditions at ψ=0:

$$e_{co}\left(d\right)=p+\frac{{e_{co}}^{{}^{\prime \prime }}\left(0\right)}{2}d^{2}$$

$${e_{co}}^{{}^{\prime }}\left(d\right)={e_{co}}^{{}^{\prime \prime }}\left(0\right)d,$$

where the ψ term has been replaced where appropriate with the infinitesimal variable *d* to avoid singular terms:

$${e_{co}}^{{}^{\prime \prime }}\left(0\right)\approx -\left(k^{2}\left(n{\left(0\right)}^{2}-{n_{co}}^{2}\right)+\frac{V^{2}}{{a_{core}}^{2}}\left(1-b\right)-\frac{l^{2}}{{a_{core}}^{2}d^{2}}\right)\frac{{a_{core}}^{2}}{2}p,$$

and $p=e_{co}\left(0\right)$ is an unknown parameter that must be solved for.

Furthermore, the slope and value of $e_{co}$ and $e_{cl}$ at ψ=1 and ψ=0 respectively should be equal:

$${e_{co}}^{{}^{\prime }}\left(1\right)={e_{cl}}^{{}^{\prime }}\left(0\right)$$

$$e_{co}\left(1\right)=e_{cl}\left(0\right)=E_{l},$$

where $E_{l}$ is an arbitrary amplitude coefficient that determines the power input into the fiber. The remaining boundary conditions are set at ψ=1 for the slope ${e_{cl}}^{{}^{\prime }}$ as r→∞:

$${e_{cl}}^{{}^{\prime }}\left(1\right)=0,$$

provided that a sufficiently large value for the cladding diameter $b_{clad}$ is chosen to approximate its end as ∞.

The problem contains two unknown parameters (the generalized guide index *b* and the parameter *p*) along with two sorder differential equations requiring a total of six boundary conditions, which are now summarized below:

(13) $$\begin{array}{c} e_{co}\left(d\right)=p+\frac{{e_{co}}^{{}^{\prime \prime }}\left(0\right)}{2}d^{2} \end{array}$$

(14) $$\begin{array}{c} {e_{co}}^{{}^{\prime }}\left(d\right)={e_{co}}^{{}^{\prime \prime }}\left(0\right)d \end{array}$$

(15) $$\begin{array}{c} e_{co}\left(1\right)=e_{cl}\left(0\right) \end{array}$$

(16) $$\begin{array}{c} e_{co}\left(1\right)=E_{l} \end{array}$$

(17) $$\begin{array}{c} {e_{co}}^{{}^{\prime }}\left(1\right)={e_{cl}}^{{}^{\prime }}\left(0\right) \end{array}$$

(18) $$\begin{array}{c} {e_{cl}}^{{}^{\prime }}\left(1\right)=0\;. \end{array}$$

The boundary value problem is also summarized below and written in terms of the elements of the state vector $\vec{q}$, the core radius $a_{core}$, the cladding radius $b_{clad}$, the core RI $n_{co}$, the cladding RI $n_{cl}$, the generalized frequency k=2π/λ and the modal parameter $V=ka_{core}\sqrt{n_{co}^{2}-n_{cl}^{2}}$. The model is in terms of normalized radial coordinates $\psi =r/a_{core}$ and $\Psi =\psi \left(b_{clad}-a_{core}\right)+a_{core}$. Here, *l* is the azimuthal mode number, which determines the mode configuration being solved for, and $E_{l}$ is an arbitrary amplitude constant that determines the power carried by an individual mode.

(19) $$\begin{array}{c} \vec{q}=\left[\begin{array}{c} e_{co}\\ \frac{de_{co}}{dr}\\ e_{cl}\\ \frac{de_{cl}}{dr} \end{array}\right]=\left[\begin{array}{c} e_{co}\\ \frac{1}{a_{core}}\frac{de_{co}}{d\psi }\\ e_{cl}\\ \frac{1}{b_{clad}-a_{core}}\frac{de_{cl}}{d\psi } \end{array}\right] \end{array}$$

(20) $$\begin{array}{c} \nabla \vec{q}=\left[\begin{array}{c} a_{core}\vec{q}\left[2\right]\\ -\frac{1}{\psi }\vec{q}\left[2\right]-\left(k^{2}\left(n{\left(r\right)}^{2}-{n_{co}}^{2}\right)+\frac{V^{2}}{{a_{core}}^{2}}\left(1-b\right)-\frac{l^{2}}{{a_{core}}^{2}\psi^{2}}\right)a_{core}\vec{q}\left[1\right]\\ \left(b_{clad}-a_{core}\right)\vec{q}\left[4\right]\\ -\frac{\left(b_{clad}-a_{core}\right)}{\Psi }\vec{q}\left[4\right]+\left(k^{2}\left(n{\left(r\right)}^{2}-{n_{cl}}^{2}\right)-\frac{V^{2}}{{a_{core}}^{2}}b-\frac{l^{2}}{\Psi^{2}}\right)\left(b_{clad}-a_{core}\right)\vec{q}\left[3\right] \end{array}\right]\;. \end{array}$$

Now that the numerical model is established, it is solved using MATLAB’s built-in boundary value solver, which utilized three-stage Lobatto methods to obtain the solution. The results of the analytical solution will be used as a guess for the numerical model in order to initialize the solution. The numerical approach is verified against the known step index solution [21,22,23] for the case of step index profiles to check the accuracy of the numerical model.

#### 2.2.3. Analytical Solutions to Step Index Profile Problems

Step index fibers feature constant core and cladding RIs with a step drop to the cladding RI. An analytical solution exists for this type of fiber, which is obtained by solving Equations (7) and (9) directly by making the substitution of $n\left(r\right)=n_{co}$ for the core region equation and $n\left(r\right)=n_{cl}$ for the cladding region producing the following well-known results [21]:

(21) $$\begin{array}{c} e_{co}\left(r\right)=E_{l}\frac{J_{l}\left(V\sqrt{1-b}\;\frac{r}{a_{core}}\right)}{J_{l}\left(V\sqrt{1-b}\right)} \end{array}$$

(22) $$\begin{array}{c} e_{cl}\left(r\right)={E_{l}}^{{}^{\prime }}\frac{K_{l}\left(V\sqrt{b}\;\frac{r}{a_{core}}\right)}{K_{l}\left(V\sqrt{b}\right)}\;, \end{array}$$

where $J_{l}$ is the Bessel function of the first kind, $K_{l}$ is the modified Bessel function of the second kind and $E_{l}$ and ${E_{l}}^{{}^{\prime }}$ are amplitude constants to be determined through boundary conditions. The result of applying the continuity boundary condition at the core/cladding boundary yields the following results for the amplitude coefficients and the generalized guide index *b*:

(23) $$\begin{array}{c} E_{l}={E_{l}}^{{}^{\prime }} \end{array}$$

(24) $$\begin{array}{c} Ba_{core}\frac{J_{l+1}\left(Ba_{core}\right)}{J_{l}\left(Ba_{core}\right)}=V^{2}-{\left(Ba_{core}\right)}^{2}\frac{K_{l+1}\left(V^{2}-{\left(Ba_{core}\right)}^{2}\right)}{K_{l}\left(V^{2}-{\left(Ba_{core}\right)}^{2}\right)}\;, \end{array}$$

where $B=V{/}_{a_{core}}\sqrt{\left(1-b\right)}$.

Next, the solutions to Maxwell’s equations are verified for the developed numerical model in Section 2.2.2 against the theoretical model in Section 2.2.3.

### 2.3. Verification of Developed Numerical Model against Step Index Solutions

Both models will be solved for the fiber presented below as it is similar to the application described in [15]. The parameters used for the case study are summarized in Table 1.

First a step index fiber is assumed and is compared against the numerical arbitrary index profile solver where n(r) is fed into the solver as a constant RI in the core and cladding regions for a few selected modes that can propagate in the fiber.

**Figure 2:**
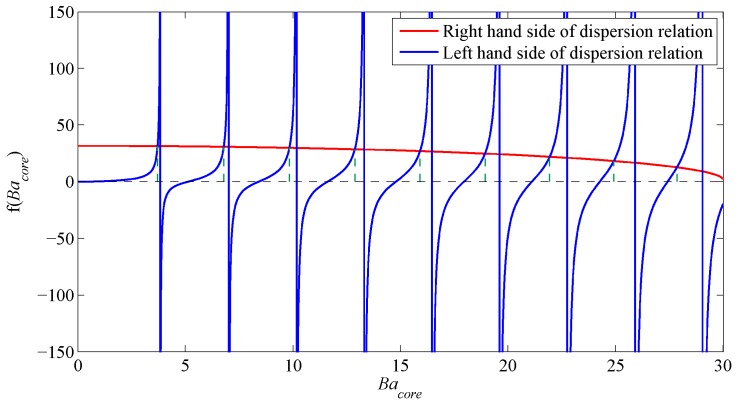
Analytical dispersion results for step index fibers (Table 1), l=1.

First, the characteristic equation, Equation (24), is solved for *b*. It cannot be solved analytically and must therefore be solved graphically by plotting the right-hand side versus the left-hand side to obtain all the possible intersections and, hence, obtain a value for the parameter *B* from which *b* may be inferred. The results for the analytical dispersion relation for the fiber above for l=1, l=2 and l=5 is solved for and used as an initial guess for the generalized guide index *b* to be input into the solver. An example of the solution is shown in Figure 2 for l=1.

Next, both the analytical solver, Equations (21) and (22), and the numerical solver, Equations (13)–(20), are run simultaneously for the following linearly-polarized modes; $LP_{1,5}$,$LP_{2,2}$ and $LP_{5,4}$ where $LP_{l,m}$ is the designation for a linearly-polarized mode with azimuthal number *l* and radial mode number *m*. A radial mode number of m=1 would correspond to the first mode solution beginning from the left of the abscissa. Furthermore, a value of $E_{l}=1$ is used in both models in order to normalize the result at the cladding/core interface. The amplitude constant $E_{l}$ is set based on how much power is coupled into a specific mode. This parameter will later on be used to distribute the input power to a fiber among the various possible modes that can be accommodated.

**Figure 3:**
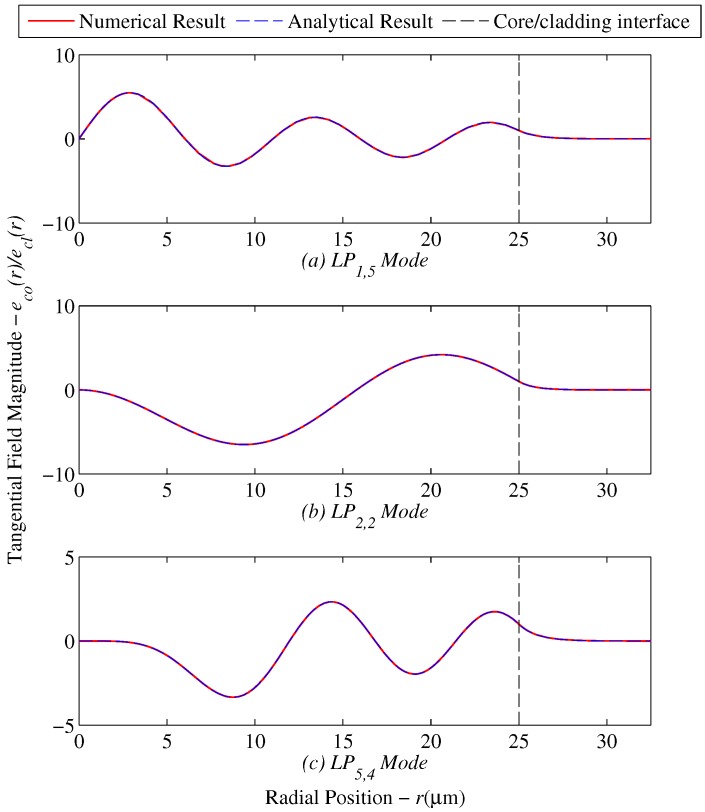
Radial field comparison for step index fibers for selected modes (**a**) $LP_{11}$, (**b**) $LP_{22}$ and (**c**) $LP_{54}$.

The results of the these comparisons are shown in Figure 3. The value for the propagation constant *b* is obtained by using the analytical dispersion solution as an initial guess. Since for this case both methods are being used to solve the same case for a step index fiber, the same value of *b* is expected from the numerical search approach. Table 2 below summarizes and compares the results of both cases for each generalized guide index.

The maximum percentage difference with reference to the analytical results for *b* is in the order of $10^{-3}$, which is expected since the numerical solver is already at the convergence point.

After gaining confidence in the numerical model, the solutions to Maxwell’s equations developed in Section 2.2 will be used to calculate the light intensity profiles in the fiber before and after contact with hydrocarbons. The results will be compared with those presented in [15], which conducted OTDR experiments on different hydrocarbons characterized by different RIs.

## 3. Characterization of OTDR Traces Using Established Theoretical and Numerical Models

In order to verify the developed theoretical tool, the attenuation results from [15] will be used. The developed theoretical tools for calculating the intensity distribution will be used to calculate the net intensity profile before and, after contact with hydrocarbons, calculate the net power and the corresponding attenuation coefficient, which will be compared against the attenuation results reported in the literature. Table 3 summarizes the properties of the fiber optic cable used for the experimental setup in [15].

A test was carried out assuming that the chemical in contact with the fiber was trichloromethane (CHCl${}_{3}$) with an RI of 1.4459. Two possible scenarios will be explored for the possible RI distributions due to soaking in CHCl${}_{3}$.

### 3.1. Refractive Index Perturbation Mechanisms

**Figure 4:**
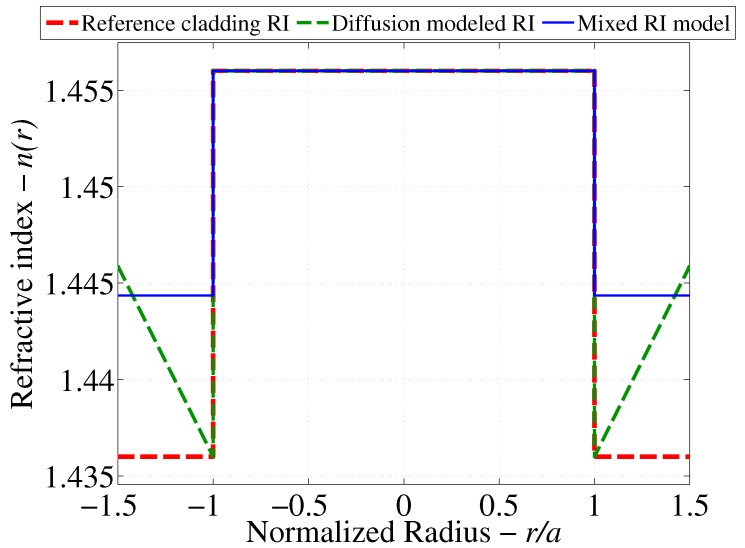
Possible RI profiles as a result of the soaking of PDMS-clad fibers in CHCl${}_{3}$.

The first scenario is considered by assuming a first order diffusion model of the hydrocarbon in question through the cladding layers. According to [24], the mass concentration of the chemical agent in question will grow in time until reaching a steady-state concentration profile that varies linearly between the initial concentration of 0 mol/L at the core/cladding interface and the ambient concentration of the hydrocarbon in the medium surrounding the cladding. This results in the diffusion modeled RI profile depicted by Figure 4.

The second scenario is considered by assuming a mixed RI as a result of combining the optical properties of the hydrocarbon in question (CHCl${}_{3}$) and the Polydimethylsiloxane (PDMS) polymer that forms the cladding. The mixed RI is predicted by calculating the volume fraction of each constituent under the assumption of complete soaking of CHCl${}_{3}$ into the PDMS cladding. Table 4 below outlines the details of the calculations used to compute the volume fraction of each constituent.

In order to calculate the RI of a mixture of PDMS and CHCl${}_{3}$ under the assumption that all the present free volume is occupied by the hydrocarbon, the generalized Lorentz–Lorenz relation [27] is adapted for this problem:

(25) $$\frac{n_{1\to m}^{2}-1}{n_{1\to m}^{2}+2}=\underset{i=1}{\sum^{m}}\;\phi_{i}\frac{n_{i}^{2}-1}{n_{i}^{2}+2}\;,$$

where $n_{1\to m}$ is the RI of *m* constituents, $n_{i}$ is the RI of each constituent and $\phi_{i}$ is the volume fraction of each constituent. Using the results from Table 3 and the established equation for the free volume fraction in PDMS, the effective cladding RI is calculated as $n_{cl}=1.44435$.

The results of the previous scenarios are summarized by Figure 4 in order to depict the cases for which the light distribution is to be solved.

### 3.2. Mode Configuration in Reference and Perturbed Fibers

**Figure 5:**
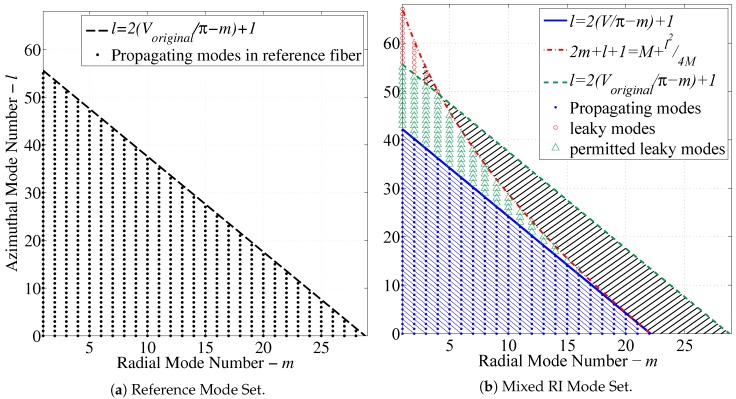
Permitted mode sets for (**a**) step index and diffusion modeled fibers and (**b**) mixed RI fibers [17].

Next, the corresponding permissible mode sets are computed in order to determine the number of modes propagating in the fiber. Each mode is represented by a combination of an azimuthal and radial mode number denoted as *l* and *m*, respectively. For each unique solution of the generalized guide index *b*, a non-negative integer value for *m* is assigned, with m=1 corresponding to the largest unique solution for *b*. Here, *l* designates the azimuthal mode number, which controls the cylindrical distribution of the radial mode solution obtained as follows:

(26) $$\begin{array}{c} e_{t}\left(r,\phi \right)=e_{co}\left(r\right)\cos l\phi \hat{x}\;for\;r\leq a_{core} \end{array}$$

(27) $$\begin{array}{c} e_{t}\left(r,\phi \right)=e_{cl}\left(r\right)\cos l\phi \hat{x}\;for\;r>a_{core} \end{array}$$

where $e_{co}$ and $e_{cl}$ are radial mode solutions in the core and cladding regions obtained from Equations (19) and (20) or the theoretical solutions [21,22,23]. The parameters *l* and *m* are enumerated and fed to the solver that is used to obtain the intensity distribution for each individual mode. The possible enumerations of the mode sets are characterized by the limiting equations for guided and leaky modes [22,28] as depicted graphically in Figure 5b.

Figure 5a displays the complete number of guided modes permitted by the fiber specified by Table 3. The same fiber under a hydrocarbon perturbation in the RI as a result of the mixed RI assumption is shown in Figure 5b. It is assumed that light traveling through the fiber is fully developed before encountering the perturbation. As a result, when the modal capacity is reduced, some modes are immediately attenuated, while others travel in the radiation domain denoted as “leaky” modes. These leaky modes are attenuated considerably faster than guided modes, but due to the short length of the perturbation, they are returned to the set of guided modes as soon as the modal capacity of the fiber is restored following the perturbation. The remainder of the modes are permanently decoupled from the fiber. This effect is represented by the black hatched area in Figure 5b. In other words, this area represents the lost energy from the fiber due to the perturbation.

### 3.3. Cumulative Light Intensity Distribution of All Possible Mode Sets

The permissible mode configurations explained in Section 3.2 can now be solved for using the developed solver in Section 2.2.2 and the analytical solution in Section 2.2.3. The solver is run for each mode while setting the amplitude constant $E_{l}$ to unity. $E_{l}$ determines the amount of power coupled into each mode relative to the other supported modes. By setting $E_{l}$ to unity for all modes, an equal power distribution among all permissible modes is enforced. In a practical test setup, this condition is met by the use of a mode scrambler to equally excite all light propagation modes [15]. The intensity distribution $e_{t}$ is recorded, and the corresponding power for each mode is calculated as: [21]

(28) $$P_{total}=\int_{0}^{2\pi }\int_{0}^{\infty }\frac{n}{\eta_{0}}{e_{t}}^{2}rdrd\phi \;.$$

All the modes are normalized to carry unity power by dividing the square of each intensity distribution ${e_{t}}^{2}$ by the total power carried as calculated in Equation (28) yielding the square of the normalized intensity distribution ${{\hat{e}}_{t}}^{2}$. The total power in the fiber is therefore given by the sum of all the normalized intensity distributions.

However, the number of modes is not simply given by the number of permissible combinations of *l* and *m* as shown in Figure 5. Examining Equations (26) and (27), for l=0, it can be seen that the cosine terms become unity. However, for l>0, the cosine terms may assume two identical states in terms of magnitude, cos(lθ) and cos(lθ+π/2) which results in each mode being counted twice due to the presence of two polarization states [28].

All supported modes for each assumption are normalized, summed and integrated by Equation (28). The ratio of the power of the perturbed fiber to the reference fiber is used to calculate the step drop per unit length (i.e., the attenuation coefficient α), which characterizes the step drop:

$$\frac{P_{perturbed}}{P_{reference}}=10^{-\alpha \Delta z/10},$$

(29) $$\alpha =-10\;log_{10}\left(\frac{P_{perturbed}}{P_{reference}}\right)/\Delta z\;,$$

where Δz is the interval of the step drop usually set to a unit meter. With the above procedure, the attenuation coefficients and respective OTDR traces can be generated for both scenarios explained in Section 3.1.

### 3.4. Power Attenuation Results for Diffusion Modeled Fibers

**Figure 6:**
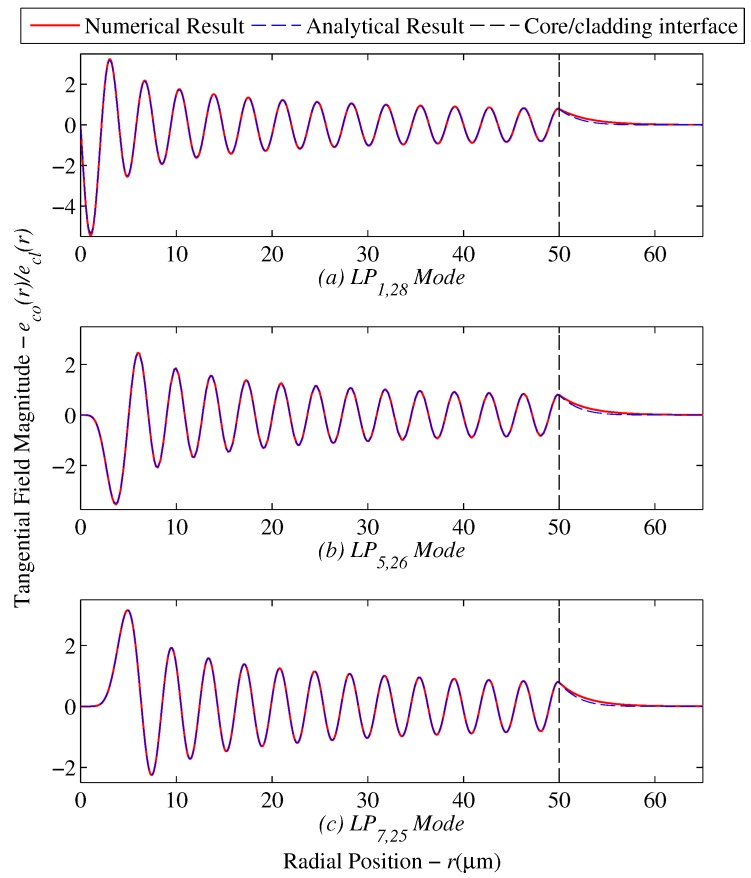
Radial field comparison for step index and diffusion modeled fibers for selected modes (**a**) $LP_{1,28}$, (**b**) $LP_{5,26}$ and (**c**) $LP_{7,25}$.

Utilizing the pre-established mode sets in Figure 5a, the numerical model for diffusion modeled clad fibers returned the intensity distributions for individual modes. Figure 6 displays the intensity results for a few selected high order modes who have strongly affected evanescent waves.

The drop in intensity is not very significant as shown in Table 5, where the maximum change in the generalized guide index was 2.98%.

**Figure 7:**
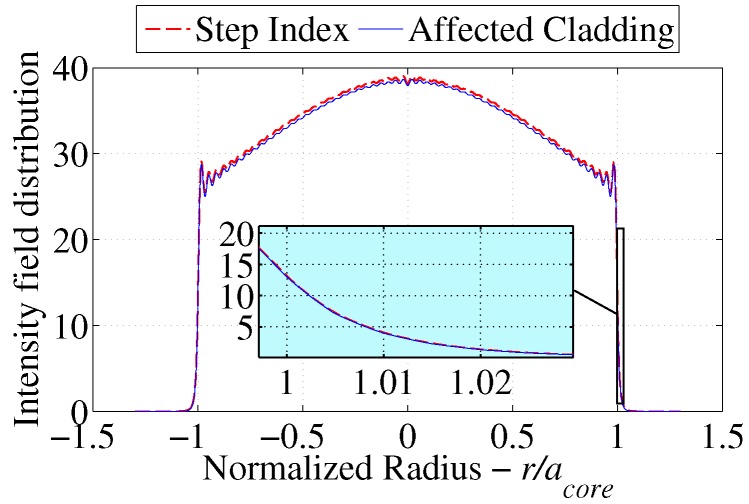
Superimposed light intensity profile comparison for reference step index fibers and diffusion modeled clad fibers.

As a result the cumulative intensity distribution is not likely to show a significant drop in power as shown in Figure 7 where the net intensity distribution slightly drifted from that of the reference step index fiber. The resulting change in power will be translated into an attenuation coefficient and compared with the results of Section 3.5.

### 3.5. Power Attenuation Results for Mixed Refractive Index Fibers

**Figure 8:**
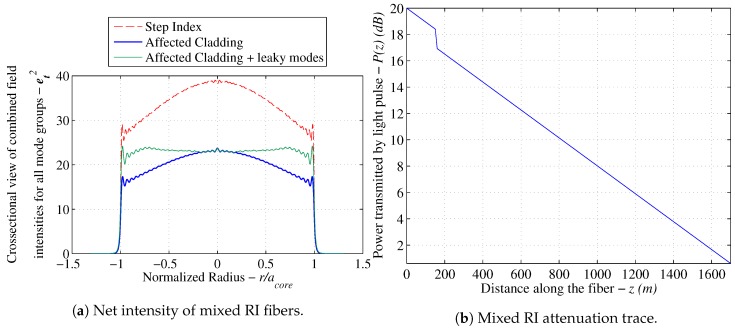
Simulation results for mixed RI fibers showing (**a**) the cumulative intensity distribution and (**b**) the expected attenuation (OTDR) trace for the calculated step drop [17].

Utilizing the pre-established mode sets in Figure 5b, the analytical solution for step index fibers is used to obtain the light intensity solution for each mode and uses the summation algorithm from Section 3.3 to obtain the cumulative light intensity distribution. The total results are reported in Figure 8. The attenuation coefficient is then reported relative to the reference fiber and summarized in Section 3.6.

### 3.6. Comparison and Discussion of Diffusion Modeled and Mixed RI Fibers as Describing Models of CHCl${}_{3}$ Perturbation

A comparison between diffusion modeled fibers and mixed RI fibers is performed to determine which of the two models best describes the reference case obtained through experiments in the literature. As noted in Section 3.4 earlier, the diffusion model is likely to yield unrealistic results due to the assumption that the RI at the core/cladding boundary is fixed. Table 6 displays this result showing a very small comparative step drop in power levels.

Mixed RI modeling exhibits more promising results as the step drop experienced due to the modeled RI profile closely agrees with the value reported in the literature of 1.46 dBm${}^{-1}$ resulting in more credibility being awarded to the mixed RI model. Due to the close agreement in the results between the reference experimental case and the mixed RI model, a calibration curve for the proposed sensor and the variation of the step drop with various liquids with various RIs will be studied by using the mixed RI model to calculate the step drop for different hydrocarbons.

## 4. Sensor Calibration Data and Discussion of Sensor Operation for Various Hydrocarbon Stimulants

**Figure 9:**
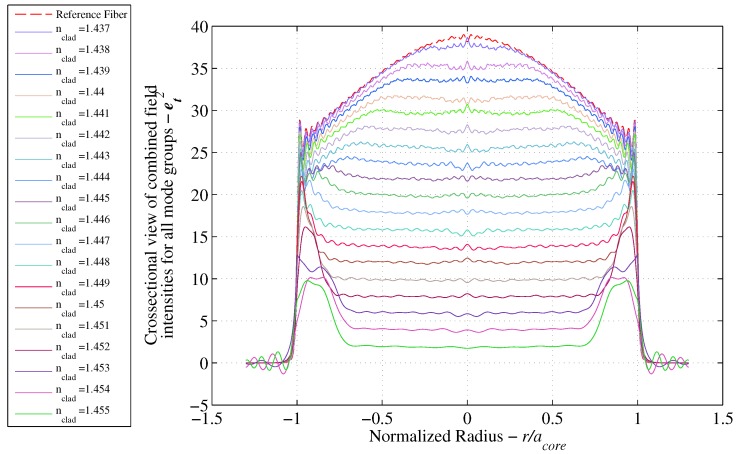
Cumulative intensity of selected cladding RI.

**Figure 10:**
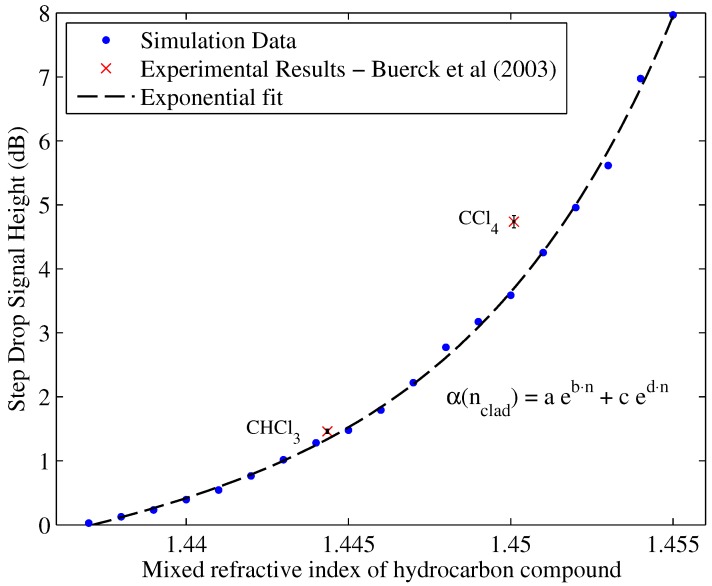
Expected step drop signal for various cladding RIs in comparison with experimental results [15].

Using the pre-established mixed RI model, a calibration curve describing the proposed sensor was sought by solving the model for selected RIs at 5% intervals. The resulting step drops computed from the cumulative light intensity distributions for each cladding interval are depicted in Figure 10, while the intensity profiles at each increment are shown in Figure 9. To assess the level of agreement with experimental data [15], the instrument used for obtaining the OTDR traces (TFS 3031, Tektronix) [15] was investigated in order to obtain a measure of uncertainty in the results.

### Design Stage Uncertainty of the Tektronix OTDR Unit

The manufacturer specifications and the uncertainty characteristics of the (TFS 3031, Tektronix) instrument are given in the manufacturer datasheets. Relevant parameters are summarized in Table 7. As a result, the uncertainty in the measurement is given as follows:

For the case of a step drop of 1.46 dB, the total design stage uncertainty is given by:

$${U_{d}}_{T}={{U_{d}}_{S}}^{2}+{{U_{d}}_{D}}^{2}={{U_{d}}_{S}}^{2}+{e_{1}}^{2}+{e_{2}}^{2}=\pm 0.0309\mathrm{dB}$$

The resulting uncertainty is incorporated into the experimental measurements to verify whether the discrepancy between theoretical calculations and experimental measurements is simply due to hardware uncertainty. It can be seen from Figure 10 that the discrepancy is not within hardware uncertainty limits, which justifies a physical explanation in the assumptions made.

A two-term exponential fit can be used for the simulated points to obtain a theoretical calibration curve for various RIs of the form:

(30) $$\alpha \left(n\right)=ae^{b\cdot n_{clad}}+ce^{d\cdot n_{clad}}$$

where coefficients are given as: a=2.109;b=0.8399;c=−0.2702;d=−0.4631.

Note that this calibration is only valid in the range $1.470\leq n_{clad}\leq 1.4550$ where $n_{clad}$ is normalized by the mean (μ) = 1.446 and the standard deviation (σ) = 0.005627 as follows: $n_{i}=\frac{n_{clad}+\mu }{\sigma }$.

Below *n* = 1.4370, the attenuation coefficient is equal 0 dB, and above 1.4550, complete power loss occurs or, for simplicity, losses in excess of 20 dB are expected. Please note that $n_{clad}$ is calculated from Equation 25 to account for the mixing of the hydrocarbon with the PDMS cladding as demonstrated in Table 4.

## 5. Discussion of Calibration Data

Figure 10 displays a close agreement between the simulated data and the experimental data points obtained by [15]. The work in [15] tested two hydrocarbon compounds; CHCl${}_{3}$ and CCl${}_{4}$, as shown in the figure. Table 8 shows the experimental discrepancy between the simulated calibration curve and the experimental points.

There is an increasing level of discrepancy as the RI approaches the core RI. The reasons for this discrepancy could be attributed to the idealization of the assumed RI profile. However, the increasing level in discrepancy can be attributed to the increasing count of leaky modes as the gap between the core RI and the cladding/hydrocarbon mixed RI diminishes.

**Figure 11:**
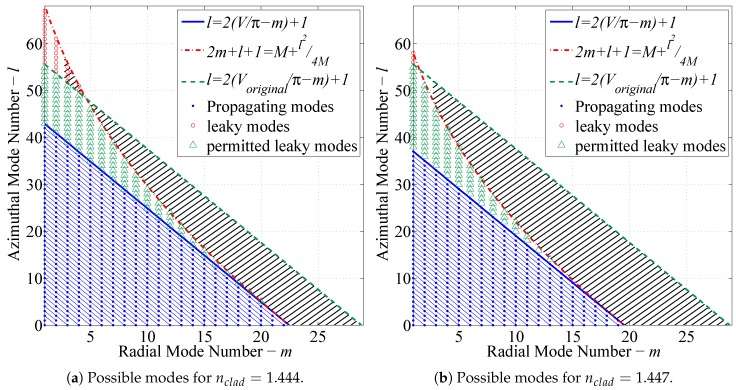
Comparison of permissible guided and leaky mode sets for (**a**) 40% of the total RI range (**b**) 60% of the total RI range.

Figure 11 demonstrates how the number of leaky modes is increased when the RI of the hydrocarbon increases. Figure 11 shows this affect as the number of leaky modes shown in green increases compared to the number of guided modes when increasing the RI from 1.444–1.447.

The attenuation coefficient calculations in this paper assume that the permitted leaky modes are loss-less and behave just like guided modes. However, that is not necessarily valid. According to [29], a leaky mode is defined as a mode that is attenuated to 1% of its original power value. This corresponds to a drop of 20 dB per meter according to Equation (29). If the perturbation length is assumed to be 1 cm, then the drop would only be 0.2 dB, which is small enough, but comparable to the calculated attenuation coefficient for CHCl${}_{3}$ of around 1.46 dB from Figure 10. In other words, the assumption that the perturbation is infinitesimal is not a valid one and must be accommodated for by studying the attenuation coefficient associated with leaky modes and incorporating them into the established mathematical model for a closer agreement.

The mathematical model provides a powerful design tool for distributed sensors that use optically-sensitive claddings to transduce an attenuation in the light pulse. Based on the optical properties of alkanes present in crude oil, 1.3571<n<1.3886 [30], the sensor needs to be redesigned to operate in this region. As a result, the model will be used to design the fiber to be used for future experimental verifications. Data obtained through material handbooks about the optical properties of several of these compounds was obtained as shown in Table 9.

**Figure 12:**
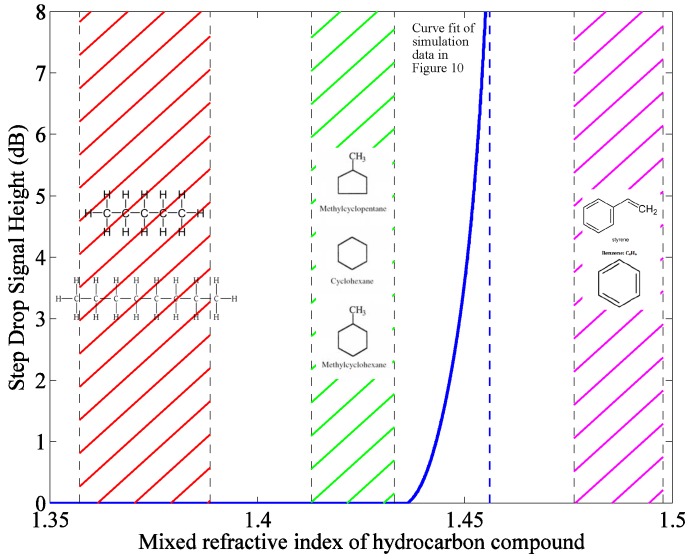
Calibration plot obtained for current sensor configuration with reference to known hydrocarbon compounds present in crude oil mixtures.

Figure 12 demonstrates the sensitivity of this sensor to crude oil and how the fiber should be redesigned for the best performance in the expected environment known to exist in oil transfer lines. A best fit line of the simulated attenuation coefficients was projected on a coordinate space showing the RI band for certain hydrocarbon species (namely those most abundant in oil and gas pipelines). The sensing fiber in its current configuration is beyond the bandwidths of any of these species (especially alkanes). As a result, when considering field deployment, the chemical composition of the pipeline environment should be known such that the core and cladding RIs are tuned to provide a detectable signal attenuation.

For example, the core RI should be set at approximately 1.38, while the cladding RI should be set at around 1.355 such that the fiber detects contact with alkanes. However, caution should be exercised when making these determinations such that the hydrocarbon compound in question does not possess an RI close to, but lower than that of the core RI. Such a situation would cause a huge attenuation in the signal rendering the remainder of the fiber insensitive as the entire signal has been stripped from the fiber.

However, if the sensing band is chosen to give a reasonable attenuation in the signal, the fiber can detect hydrocarbon intrusion at multiple points along its length allowing for spatial monitoring of the fiber condition without the need for replacement of the already exposed sections of the fiber.

## 6. Conclusions and Future Work

An analytical modeling tool was developed to simulate the performance of a hydrocarbon OTDR sensor operating as a corrosion indicator in oil pipelines. The data obtained by the tool agree with several data points from the literature with a certain measure of discrepancy due to a number of idealizations made. The modeling tool describes the proposed sensor’s performance for a variety of hydrocarbon stimulants allowing the sensor to be redesigned for operation in crude oil mixtures. It will help to provide insight into the design of the experimental setup needed to accommodate and test the sensor’s operation.

Furthermore, mathematical modeling of all the guided and leaky modes provides insight into the attenuation coefficient of each mode, which can be used to infer the length of the perturbation to be made in the sensing fiber. The step drop exhibited following a hydrocarbon perturbation should contain two components: attenuation due to loss of higher order modes and the relatively large attenuation associated with leaky modes. By being able to quantify the latter, the length traveled by the leaky modes throughout the perturbation may be inferred.

### Potential Applications of This Technology

In this paper, an optical fiber corrosion sensor with a hydrocarbon sensitive cladding, coated with a metal layer similar to the pipeline material, was introduced to detect internal corrosion of oil pipelines. In this paper, the cladding was sensitive to hydrocarbons, but the cladding can be made sensitive to other chemicals or conditions such as corrosion-causing bacteria, scale, asphaltene, wax or all the above. The potential applications of our proposed fiber optics sensor in the oil and gas industry are enormous.

## Figures and Tables

**Table 1:** Parameters characterizing the waveguide fiber used to verify solution methods. RI, Refractive Index.

Parameter	Designation	Value
Core Radius (μm)	$a_{core}$	25
Cladding Outer Radius (μm)	$b_{clad}$	50
Core RI	$n_{co}$	1.465
Cladding RI	$n_{cl}$	1.456
Wavelength of Light Pulse (nm)	λ	850
Numerical Aperture	NA	0.162
Modal Capacity-VNumber	*V*	30

**Table 2:** Comparison between the propagation constant estimates for both the analytic and numerical approaches to step index problems.

Mode Number	$b_{\mathrm{analytical}}$	$b_{\mathrm{numerical}}$	% Difference
$LP_{1,5}$	0.71868776637647	0.718668345268902	$-2.702\times 10^{-3}$
$LP_{2,2}$	0.926331802523902	0.926331803204183	$7.3438\times 10^{-8}$
$LP_{5,4}$	0.626809596890269	0.626808854980724	$-1.184\times 10^{-4}$

**Table 3:** Parameters characterizing the experimental waveguide fiber [15] to verify solution methods.

Parameter	Designation	Value
Core Radius (μm)	$a_{core}$	50
Cladding Outer Radius (μm)	$b_{clad}$	100
Core RI	$n_{co}$	1.456
Cladding RI	$n_{cl}$	1.436
Wavelength of Light Pulse (nm)	λ	850
Numerical Aperture	NA	0.24
Modal Capacity-V Number	*V*	88.89

**Table 4:** Free volume calculation of PDMS polymer utilizing established PDMS properties.

Parameter	Designation	Value
Number average molecular weight (g/mol) [25]	$M_{n}$	73,474.00
Molecular weight of monomer repeat unit (g/mol)	$m_{w}$	74.15
Density of PDMS (kg/m${}^{3}$) [26]	ρ	970.00
Diameter of monomer repeat unit (Å) [26]	$D_{m}$	3.07
Length of monomer repeat unit (Å) [26]	$l_{m}$	2.69
Volume of monomer repeat unit (Å${}^{3}$)	$V_{m}{=}^{\pi {D_{m}}^{2}l_{m}}{/}_{4}$	19.91
Volume fraction of polymer to free volume (%)	$V_{polymer}=\rho V_{m}N_{A}/m_{w}$	15.60
Free volume fraction (%)	$1-V_{polymer}$	84.40

**Table 5:** Comparison between the propagation constant estimates for both the step index and diffusion modeled clad fibers.

Mode Number	$b_{\mathrm{step}\mathrm{index}}$$\times 10^{-2}$	$b_{\mathrm{diffusion}\mathrm{clad}}$$\times 10^{-2}$	Relative Difference (%)
$LP_{1,28}$	3.4076	3.3059	2.98
$LP_{5,26}$	3.6788	3.5853	2.54
$LP_{7,25}$	3.9527	3.8666	2.18

**Table 6:** Comparison between the power transmission parameters for a reference fiber, a CHCl${}_{3}$ saturated fiber and a mixed RI fiber.

Parameter	Reference Fiber	Diffusion Modeled Fiber	Mixed RI Fiber
Number of supported modes	1596	1596	1162
Total guided power (mW)	100.016	98.72	72.9143
ine Power loss (%)		1.30	27.10
Attenuation coefficient (dBm${}^{-1}$)		0.00206	1.373
ine Reported attenuation			
Coefficient [15] (dBm${}^{-1}$)		1.46	

**Table 7:** Design stage uncertainty analysis on the TFS 3031, Tektronix Optical Time Domain Reflectometry (OTDR) unit used for experimental OTDR measurements [15].

Parameter	Designation	Value
System measurement uncertainty	${U_{d}}_{S}$	±0.01 dB
Output display accuracy	${U_{d}}_{D}$	
Readout resolution	$e_{1}$	±0.00005 dB
Vertical linearity	$l_{e}$	0.02 dB/dB
Linearity uncertainty	$e_{2}$	l×reading

**Table 8:** Comparison between experimental data points in [15] and the simulated calibration curve.

	Effective RI of HC and Cladding (Equation (25))	Expected Attenuation Drop (Simulated) dB (Equation (30))	Attenuation Drop dB Ref. [15])	Difference (%) *
First Data				
Point (CHCl${}_{3}$)	1.4444	1.3394	1.46	8.2617
Second Data				
Point (CCl${}_{4}$)	1.4501	3.6952	4.737	21.9930

* Percentage difference reported as: $\left|\frac{Attenuation_{ref}-Attenuation_{simulated}}{Attenuation_{ref}}\right|\times 100$.

**Table 9:** List of hydrocarbon compounds composing crude oil and their respective RIs.

Parameter	RI	Mixed RI with PDMS
Alkanes		
Pentane [31]	1.3430	1.3571
Octane [31]	1.3800	1.3886
Cycloalkanes (Naphthenes)		
Methylcyclopentane [32]	1.409	1.413
Cyclohexane [33]	1.4325	1.4330
Methylcyclohexane [34]	1.422	1.424
Aromatic Compounds		
Benzene [35]	1.4839	1.4763
Styrene [36]	1.5095	1.4978
Toluene [37]	1.4850	1.4772

## Supplementary Materials

The following are available online at www.mdpi.com/1424-8220/17/10/2227/s1.

## Nomenclature

$e_{t}$	Tangential component of electric field vector
$e_{co}$	Radial electric field intensity distribution in the core
$e_{cl}$	Radial electric field intensity distribution in the cladding
*l*	Azimuthal mode number
*m*	Radial mode number
ϕ	Azimuthal coordinate in cylindrical geometry
*r*	Radial coordinate in cylindrical geometry
*z*	Axial coordinate in cylindrical geometry
$a_{core}$	Core radius
$b_{clad}$	Cladding outer radius
β	Propagation constant
$\mu_{0}$	Magnetic permittivity of free space
$\varepsilon_{0}$	Electric permittivity of free space
ω	Angular velocity of light
$n\left(r\right)$	Refractive index radial profile
$\nabla^{2}$	Laplacian operator
*k*	Generalized frequency parameter f(ω)
*V*	Non-dimensional modal number
*b*	Generalized guide index
$n_{co}$	Core refractive index
$n_{cl}$	Cladding refractive index
ψ	Parametric radial coordinate transformation
$\vec{q}$	State vector
*d*	Infinitesimal coordinate near origin
*p*	Boundary value problem unknown parameter
$E_{l}$	Amplitude constant
$J_{l}$	Bessel function of the first kind
$K_{l}$	Modified Bessel function of the second kind
λ	Wavelength of light
NA	Numerical aperture
$LP_{l,m}$	Linearly polarized mode l,m
$V_{m}$	Volume of monomeric unit
$D_{m}$	Diameter of monomeric unit
$l_{m}$	Length of monomeric unit
$M_{n}$	Number average molecular weight
$m_{w}$	Molecular weight
ρ	Density of PDMS
DP	Degree of Polymerization
$m_{c}$	Mass of polymer chain
$N_{A}$	Avogadro’s constant
$N_{c}$	Number of chains
$V_{c}$	Volume of polymer chains
$m_{total}$	Total mass of fiber
$V_{polymer}$	Total volume of polymer
$\phi_{i}$	Volume fraction of constituent
$n_{1\to m}$	Mixed refractive index of m constituents
$n_{i}$	Refractive index of constituent *i*
$P_{total}$	Total guided power
$\eta_{0}$	Ratio of magnetic to electric permittivity
$P_{perturbed}$	Total guided power of perturbed fiber
$P_{reference}$	Total guided power of reference unaffected fiber
α	Attenuation coefficient
Δz	Length of perturbation
${U_{d}}_{S}$	System measurement uncertainty
${U_{d}}_{D}$	Output display accuracy
$e_{1}$	Readout Resolution
$l_{e}$	Vertical Linearity
$e_{2}$	Linearity uncertainty
UAE	United Arab Emirates
PIG	Pipeline Inspection Gage
MFL	Magnetic Flux Leakage
CCTV	Closed Circuit TV
OFS	Optical Fiber Sensors
SHM	Structural Health Monitoring
LPG	Long Period Grating
FBG	Fiber Bragg Grating
RI	Refractive Index
OTDR	Optical Time Domain Reflectometry
PDMS	Polydimethylsiloxane
ADNOC	Abu Dhabi National Oil Company
ADCO	Abu Dhabi Company for Onshore Oil Operations
