# Supplementary material for: Analytical Modeling Tool for Design of Hydrocarbon Sensitive Optical Fibers
Source: Sensors (Basel). 2017 Sep 28;17(10):2227. doi: 10.3390/s17102227 (PMC5677271; doi:10.3390/s17102227)
Supplement: Supplementary file 1 [file sensors-17-02227-s001.pdf]

Supplementary Materials: Analytical Modeling Tool for Design of Hydrocarbon Sensitive Optical Fibers

Khalil Al Handawi [1,†], Nader Vahdati [1,*], Oleg Shiryayev [1] and Lydia Lawand [1,†]

1. Formulation of Light Intensity Distribution Problem in Circular Fiber Optic Waveguides

A numerical model of the Polymer Clad Fiber (**PCS**) fiber system is developed for standard step-index fibers. Step index fibers are defined as fibers having a constant Refractive Index (**RI**) in the core region and a constant **RI** in the cladding region which is lower than that in the core region.

Electric and magnetic fields that compose the light signal vary in a sinusoidal fashion and it is therefore justifiable to use the time harmonic form of Maxwell's equations derived as follows from the time-dependent Maxwell equations:

$$\nabla \times \mathbf{E}(\mathbf{r},t) = -\mu_0 \frac{\partial \mathbf{H}(\mathbf{r},t)}{\partial t} \tag{1}$$

$$\nabla \times \mathbf{H}(\mathbf{r},t) = \varepsilon_0 \frac{\partial \mathbf{E}(\mathbf{r},t)}{\partial t} \tag{2}$$

$$\nabla \cdot \mathbf{E}(\mathbf{r},t) = 0 \tag{3}$$

$$\nabla \cdot \mathbf{H}(\mathbf{r},t) = 0 \tag{4}$$

Where μ_0 and ε_0 are the magnetic and electrical permittivities of free space respectively and $\mathbf{E}(\mathbf{r},t)$ and $\mathbf{H}(\mathbf{r},t)$ are the electric and magnetic fields respectively. r is the radial coordinate and t is time. It can be deduced that:

$$\mathbf{E}(\mathbf{r},t) = \mathbf{E}(\mathbf{r},\omega)\, e^{j\omega t} \tag{5}$$

$$\frac{\partial \mathbf{E}(\mathbf{r},\omega)\, e^{j\omega t}}{\partial \mathrm{t}} = j\omega\, \mathbf{E}(\mathbf{r},\omega)\, e^{j\omega t} \tag{6}$$

When converting a time-dependent signal to a time-harmonic representation in terms of the angular frequency of light ω. Substituting Equation (5) and Equation (6) into Equations (1) through (4), the time harmonic Maxwell equations are obtained:

$$\nabla \times \mathbf{E}(\mathbf{r},\omega) = -j\omega\mu_0 \mathbf{H}(\mathbf{r},\omega) \tag{7}$$

$$\nabla \times \mathbf{H}(\mathbf{r},\omega) = j\omega\varepsilon_0 \mathbf{E}(\mathbf{r},\omega) \tag{8}$$

$$\nabla \cdot \mathbf{E}(\mathbf{r},\omega) = 0 \tag{9}$$

$$\nabla \cdot \mathbf{H}(\mathbf{r},\omega) = 0 \tag{10}$$

Next the, constraints of traveling guided waves are introduced in order to obtain the governing relations. The assumption that the **RI** in the direction of wave propagation (the z-direction) does not vary is made. However, it may vary transversely in the x and y directions. We hence separate the fields into transverse ($\mathbf{e}_t$,$\mathbf{h}_t$) and longitudinal (e_z,h_z) fields as follows:

$$\mathbf{E}(\mathbf{r},\omega) = [\mathbf{e}_t(x,\ y) + \hat{\mathbf{z}} e_z(x,\ y)]\, e^{-j\beta z} \tag{11}$$

$$\mathbf{H}(\mathbf{r},\omega) = [\mathbf{h}_t(x,\ y) + \hat{\mathbf{z}} h_z(x,\ y)]\, e^{-j\beta z} \tag{12}$$

Where β is a propagation constant that needs to be determined. Substituting Equation (11) and Equation (12) into Equation (7) and Equation (8) where ($\nabla = \nabla_t - j\beta\hat{\mathbf{z}}$) and $n(x,y)$ is the refractive index transverse profile:

$$\nabla_t \times \mathbf{e}_t(x,y) - j\beta\hat{\mathbf{z}} \times \mathbf{e}_t(x, y) + \nabla_t e_z(x, y) \times \hat{\mathbf{z}} = -j\omega\mu_0(\mathbf{h}_t(x, y) + \hat{\mathbf{z}}h_z(x, y)) \tag{13}$$

$$\nabla_t \times \mathbf{h}_t(x,y) - j\beta\hat{\mathbf{z}} \times \mathbf{h}_t(x, y) + \nabla_t h_z(x, y) \times \hat{\mathbf{z}} = j\omega\varepsilon_0 n^2(x,y)(\mathbf{e}_t(x, y) + \hat{\mathbf{z}}e_z(x, y)) \tag{14}$$

Equations (13) and (14) can be split into equations in the $x - y$ directions and the z direction as follows:

$$-j\beta\hat{\mathbf{z}} \times \mathbf{e}_t(x, y) + \nabla_t e_z(x, y) \times \hat{\mathbf{z}} = -j\omega\mu_0\mathbf{h}_t(x, y) \tag{15}$$

$$\nabla_t \times \mathbf{e}_t(x,y) = -j\omega\mu_0 h_z(x, y)\hat{\mathbf{z}} \tag{16}$$

$$-j\beta\hat{\mathbf{z}} \times \mathbf{h}_t(x, y) + \nabla_t h_z(x, y) \times \hat{\mathbf{z}} = j\omega\varepsilon_0 n^2(x,y)\mathbf{e}_t(x, y) \tag{17}$$

$$\nabla_t \times \mathbf{h}_t(x,y) = j\omega\varepsilon_0 n^2(x,y)e_z(x, y)\hat{\mathbf{z}} \tag{18}$$

Knowing that $\nabla_t e_z(x, y) \times \hat{\mathbf{z}} = -\hat{\mathbf{z}} \times \nabla_t e_z(x, y)$, the equations for a wave guide can finally be expressed as:

$$-j\beta\hat{\mathbf{z}} \times \mathbf{e}_t(x, y) - \hat{\mathbf{z}} \times \nabla_t e_z(x, y) = -j\omega\mu_0\mathbf{h}_t(x, y) \tag{19}$$

$$\nabla_t \times \mathbf{e}_t(x,y) = -j\omega\mu_0 h_z(x, y)\hat{\mathbf{z}} \tag{20}$$

$$-j\beta\hat{\mathbf{z}} \times \mathbf{h}_t(x, y) - \hat{\mathbf{z}} \times \nabla_t h_z(x, y) = j\omega\varepsilon_0 n^2(x,y)\mathbf{e}_t(x, y) \tag{21}$$

$$\nabla_t \times \mathbf{h}_t(x,y) = j\omega\varepsilon_0 n^2(x,y)e_z(x, y)\hat{\mathbf{z}} \tag{22}$$

The major assumption made in linearly polarized weakly guiding fibers is that one of the transverse Cartesian components is much greater than the other i.e ($\mathbf{h_t} \approx 0$ in the case of transverse electric modes).

The second assumption is that the field varies smoothly in the transverse direction when compared to the longitudinal direction. This is expressed as:

$$\nabla_t e_z(x, y) \ll \beta\mathbf{e}_t(x, y)$$

$$\nabla_t h_z(x, y) \ll \beta\mathbf{h}_t(x, y)$$

Using the above assumptions, Equation (19) can be rewritten as:

$$-j\beta\hat{\mathbf{z}} \times \mathbf{e}_t(x, y) \approx -j\omega\mu_0\mathbf{h}_t(x, y)$$

$$\mathbf{h}_t(x, y) \approx \frac{\beta}{\omega\mu_0}\hat{\mathbf{z}} \times \mathbf{e}_t(x, y) \tag{23}$$

Similarly, Equation (21) can be written as:

$$-j\beta\hat{\mathbf{z}} \times \mathbf{h}_t(x, y) \approx j\omega\varepsilon_0 n^2(x,y)\mathbf{e}_t(x, y)$$

$$\mathbf{h}_t(x, y) \times \hat{\mathbf{z}} \approx \frac{\omega\varepsilon_0 n^2(x,y)}{\beta}\mathbf{e}_t(x, y) \tag{24}$$

Using the identity of the cross-product:

$$(u \times v) \times w = (u \cdot w)v - (v \cdot w)u \rightarrow (u \times v) \times v = (u \cdot v)v - (v \cdot v)u$$

Rearranging:

$$u = \frac{(u \cdot v)v - (u \times v) \times v}{(v \cdot v)}$$

For the case $u = \mathbf{h}_t = \begin{bmatrix} h_{t1} \\ h_{t2} \\ 0 \end{bmatrix}$ and $v = \hat{\mathbf{z}} = \begin{bmatrix} 0 \\ 0 \\ 1 \end{bmatrix}$, $(u \cdot v) = 0$ since both vectors are orthogonal and $(v \cdot v) = 1$ since $\hat{\mathbf{z}}$ is a unit vector:

$$u = -(u \times v) \times v = v \times (u \times v)$$

Hence by rearranging Equation (24) using the above:

$$\mathbf{h}_t(x, y) \approx \frac{\omega \varepsilon_0 n^2(x, y)}{\beta} \hat{\mathbf{z}} \times \mathbf{e}_t(x, y) \tag{25}$$

Equations (23) and (24) hold if and only if:

$$\frac{\omega \varepsilon_0 n^2(x, y)}{\beta} = \frac{\beta}{\omega \mu_0}$$

$$\beta^2 = \omega^2 \mu_0 \varepsilon_0 n^2(x, y) \rightarrow \beta = \omega n(x, y) \sqrt{\mu_0 \varepsilon_0} = kn(x, y)$$

where $k = \omega \sqrt{\mu_0 \varepsilon_0}$

Looking at Equation (23), $\frac{\beta}{\omega \mu_0} = \frac{\omega n(x, y) \sqrt{\mu_0 \varepsilon_0}}{\omega \mu_0} = {}^{n(x,y)}/_{\eta_0}$ where $\eta_0 = \sqrt{{}^{\mu_0}/_{\varepsilon_0}}$

Hence:

$$\mathbf{h}_t(x, y) \approx \frac{n(x, y)}{\eta_0} \hat{\mathbf{z}} \times \mathbf{e}_t(x, y) \tag{26}$$

Now that the relation between $\mathbf{h}_t(x, y)$ and $\mathbf{e}_t(x, y)$ has been established, the other field components can be expressed in terms of $\mathbf{e}_t(x, y)$.

From Equation (20) we can deduce the following:

$$\hat{\mathbf{z}} \cdot \nabla_t \times \mathbf{e}_t(x, y) = -j\omega\mu_0 h_z(x, y)$$

$$\hat{\mathbf{z}} \cdot \nabla_t \times \mathbf{e}_t(x, y) = -j\omega\mu_0 h_z(x, y)$$

Knowing that $j = -1/j$

$$h_z(x, y) \approx \frac{j}{\omega \mu_0} \hat{\mathbf{z}} \cdot \nabla_t \times \mathbf{e}_t(x, y) \tag{27}$$

Similarly, by substituting Equation (26) in Equation (22) we can deduce the following:

$$\nabla_t \times \mathbf{h}_t(x, y) = j\omega\varepsilon_0 n^2(x, y) e_z(x, y) \hat{\mathbf{z}}$$

$$\nabla_t \times \frac{n(x,y)}{\eta_0} \hat{\mathbf{z}} \times \mathbf{e}_t(x, y) = j\omega\varepsilon_0 n^2(x, y) e_z(x, y) \hat{\mathbf{z}}$$

$$\nabla_t \times \hat{\mathbf{z}} \times \mathbf{e}_t(x, y) = j\beta e_z(x, y) \hat{\mathbf{z}}$$

Using the identity $\mathbf{a} \times (\mathbf{b} \times \mathbf{c}) = \mathbf{b}(\mathbf{a} \cdot \mathbf{c}) - \mathbf{c}(\mathbf{a} \cdot \mathbf{b})$:

$$\nabla_t \times \hat{\mathbf{z}} \times \mathbf{e}_t(x, y) = \hat{\mathbf{z}}(\nabla_t \cdot \mathbf{e}_t(x, y)) - \mathbf{e}_t(x, y)(\nabla_t \cdot \hat{\mathbf{z}}) \text{ where } \nabla_t \cdot \hat{\mathbf{z}} = 0$$

$$\hat{\mathbf{z}}(\nabla_t \cdot \mathbf{e}_t(x, y)) = j\beta e_z(x, y) \hat{\mathbf{z}}$$

$$\nabla_t \cdot \mathbf{e}_t(x, y) = j\beta e_z(x, y)$$

$$e_z(x, y) = -\frac{j}{\beta} \nabla_t \cdot \mathbf{e}_t(x, y) \tag{28}$$

From the above field Equations (26), (27) and (28) it can be seen that the only unknown variable in the case of transverse electric field fibers (where transverse magnetic fields, $\mathbf{h}_t \approx 0$) is $\mathbf{e}_t$ from which all other field components can be determined.

The light intensity field problem is given in terms of the solution for this unknown transverse electric field $\mathbf{e}_t$. The following discussion provides two theoretical methods that are well established for two kinds of fibers featuring a step index profile and a graded index profile which will be explained as part of the discussion. A numerical method is then developed and verified against the two solutions to test its validity when applied to the fiber in question used for the detection of hydrocarbon environments.

2. General Index profile problem definition

The governing differential equation for a fiber with an arbitrary index profile will be derived first. The principle behind its derivation is that each component of the electric $\mathbf{E}(\mathbf{r},t)$ and magnetic $\mathbf{H}(\mathbf{r},t)$ fields should satisfy Maxwell's equations including the transverse field $\mathbf{e}_t$ being solved for. The condition for satisfying Maxwell's equations is known as the wave equation which can be derived from Maxwell's equations as follows:

Taking the derivative of the first Maxwell Equation (1):

$$\nabla \times (\nabla \times \mathbf{E}(\mathbf{r},t)) = -\mu_0 \left(\frac{\partial}{\partial t} \nabla \times \mathbf{H}(\mathbf{r},t) \right)$$

Using the identity: $\nabla \times (\nabla \times \mathbf{E}) \equiv \nabla (\nabla \cdot \mathbf{E}) - \nabla^2 \mathbf{E}$ where ∇^2 is the Laplacian operator

$$\nabla (\nabla \cdot \mathbf{E}) - \nabla^2 \mathbf{E} = -\mu_0 \left(\frac{\partial}{\partial t} \nabla \times \mathbf{H} \right)$$

From Equation (3):

$$\nabla \cdot \mathbf{E} = 0$$

$$\nabla^2 \mathbf{E} = \mu_0 \left(\frac{\partial}{\partial t} \nabla \times \mathbf{H} \right)$$

Substituting Equation (2) in the previous equation yields:

$$\nabla^2 \mathbf{E} = \mu_0 \varepsilon_0 n^2 \left(\frac{\partial^2 \mathbf{E}}{\partial t^2} \right)$$

$$\nabla^2 \mathbf{E} - \mu_0 \varepsilon_0 n^2 \left(\frac{\partial^2 \mathbf{E}}{\partial t^2} \right) = 0 \qquad (29)$$

Equation (29) is commonly known as the wave equation in the time domain. In the frequency domain, it is derived by using Equation (5) and (6) on Equation (29).

$$\nabla^2 \mathbf{E}(\mathbf{r},\omega) e^{j\omega t} - \mu_0 \varepsilon_0 n^2 \left((j\omega)^2 \mathbf{E}(\mathbf{r},\omega) e^{j\omega t} \right) = 0$$

$$\nabla^2 \mathbf{E}(\mathbf{r},\omega) e^{j\omega t} + \mu_0 \varepsilon_0 \omega^2 n^2 \left(\mathbf{E}(\mathbf{r},\omega) e^{j\omega t} \right) = 0$$

$$\nabla^2 \mathbf{E}(\mathbf{r},\omega) + \mu_0 \varepsilon_0 \omega^2 n^2 \mathbf{E}(\mathbf{r},\omega) = 0 \qquad (30)$$

Substituting the definition of a guided wave given by Equation (11), $\mathbf{E}(\mathbf{r},\omega) = [\mathbf{e}_t(x, y) + \hat{\mathbf{z}} e_z(x, y)] e^{-j\beta z}$:

$$\nabla^2 [\mathbf{e}_t(x, y) + \hat{\mathbf{z}} e_z(x, y)] e^{-j\beta z} + \mu_0 \varepsilon_0 \omega^2 n^2 [\mathbf{e}_t(x, y) + \hat{\mathbf{z}} e_z(x, y)] e^{-j\beta z} = 0$$

Considering only the transverse components of the electric field:

$$\nabla^2 [\mathbf{e}_t(x, y)] e^{-j\beta z} + \mu_0 \varepsilon_0 \omega^2 n^2 [\mathbf{e}_t(x, y)] e^{-j\beta z} = 0 \qquad (31)$$

Where ∇^2 is the laplacian operator defined as:

$$\left[\frac{1}{r}\frac{\partial}{\partial r}\left(r\frac{\partial}{\partial r}\right) \quad \frac{1}{r^2}\left(\frac{\partial^2}{\partial \phi^2}\right) \quad \frac{\partial^2}{\partial z^2}\right]$$

Equation (30) is known as the Helmholtz equation. By considering the above Helmholtz equation that is in terms of the transverse components, a form of the solution for $\mathbf{e}_t$ must be assumed in order to apply the Laplacian operator to the components. From the earlier definition of the fields varying sinusoidally around the axis of the fiber; i.e there is a sinusoidal variation in the field in the azimuthal direction ϕ as defined by the following Figure S1:

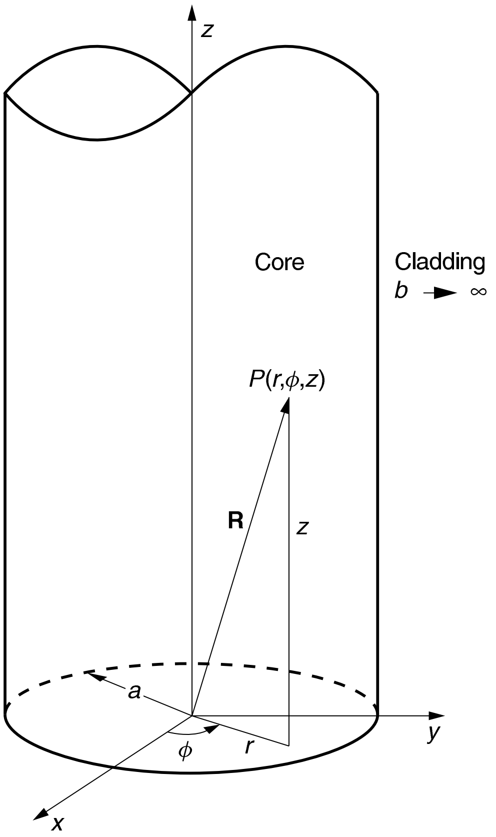

Figure S1. Optical fiber in a cylindrical coordinate system [1]

The assumed form of the solution for $\mathbf{e}_t$ is as follows in the core and cladding regions respectively:

$$\mathbf{e}_t\left(r,\phi\right) = e_{co}\left(r\right)\cos l\phi\hat{\mathbf{x}} \; for \; r \leq a_{core} \tag{32}$$

$$\mathbf{e}_t\left(r,\phi\right) = e_{cl}\left(r\right)\cos l\phi\hat{\mathbf{x}} \; for \; r > a_{core} \tag{33}$$

Where r,ϕ and l are the radial distance, the azimuthal angle and the azimuthal number defining the mode designation of the different possible modes for each non-negative integer value of $l = 0, 1, 2....$ e_{co} and e_{cl} are the radial electric field intensity solutions in the core and cladding regions respectively.

substituting the assumed solutions; Equations (32) and (33) into Equation (31):

$$\frac{1}{r}\frac{\partial}{\partial r}\left(r\frac{\partial \mathbf{e}_t\left(r,\phi\right)}{\partial r}\right)e^{-j\beta z} + \frac{1}{r^2}\left(\frac{\partial^2 \mathbf{e}_t\left(r,\phi\right)}{\partial \phi^2}\right)e^{-j\beta z} + \mathbf{e}_t\left(r,\phi\right)\left(j\beta\right)^2 e^{-j\beta z} + \mu_0\varepsilon_0\omega^2 n^2 \mathbf{e}_t\left(r,\phi\right)e^{-j\beta z} = 0$$

$$\frac{1}{r}\frac{\partial}{\partial r}\left(r\frac{\partial}{\partial r}e_{co}\left(r\right)\cos l\phi\right) + \frac{1}{r^2}\left(\frac{\partial^2}{\partial \phi^2}e_{co}\left(r\right)\cos l\phi\right) + e_{co}\left(r\right)\cos l\phi(j\beta)^2 + \mu_0\varepsilon_0\omega^2 n^2 e_{co}\left(r\right)\cos l\phi = 0$$

$$\frac{1}{r}\frac{\partial}{\partial r}\left(r\frac{\partial e_{co}\left(r\right)}{\partial r}\right)\cos l\phi - \frac{l^2}{r^2}e_{co}\left(r\right)\cos l\phi - e_{co}\left(r\right)\beta^2\cos l\phi + \mu_0\varepsilon_0\omega^2 n^2 e_{co}\left(r\right)\cos l\phi = 0$$

$$\frac{1}{r}\frac{\partial}{\partial r}\left(r\frac{\partial e_{co}(r)}{\partial r}\right)-\frac{l^2}{r^2}e_{co}(r)-e_{co}(r)\beta^2+\mu_0\varepsilon_0\omega^2 n^2 e_{co}(r)=0$$
$$\frac{1}{r}\frac{\partial}{\partial r}\left(r\frac{\partial e_{co}(r)}{\partial r}\right)+\left(\mu_0\varepsilon_0\omega^2 n^2-\beta^2-\frac{l^2}{r^2}\right)e_{co}(r)=0$$
$$\frac{1}{r}\frac{\partial}{\partial r}\left(r\frac{\partial e_{co}(r)}{\partial r}\right)+\left(k^2 n(r)^2-\beta^2-\frac{l^2}{r^2}\right)e_{co}(r)=0 \tag{34}$$

A similar approach can be followed for the derivation of the governing equation for the field in the cladding region by substituting Equation (33) into the Helmholtz equation in place of the transverse electric field component $\mathbf{e}_t$ yielding:

$$\frac{1}{r}\frac{\partial}{\partial r}\left(r\frac{\partial e_{cl}(r)}{\partial r}\right)+\left(k^2 n(r)^2-\beta^2-\frac{l^2}{r^2}\right)e_{cl}(r)=0 \tag{35}$$

Where $k^2=\mu_0\varepsilon_0\omega^2$.

according to Ref. [2], the term $k^2n^2-\beta^2$ is defined as follows for the core region:

$$k^2 {n_{co}}^2-\beta^2=\frac{V^2}{{a_{core}}^2}(1-b)$$

Where V is the modal capacity of the fiber defined as $V=ka_{core}\sqrt{{n_{co}}^2-{n_{cl}}^2}$, a_{core} is the fiber core radius and b is the unknown generalized guide index parameter which will be solved for through the application of boundary conditions to the governing equations.

Equation (34) is left in terms of $n(r)$ to accommodate an arbitrary core **RI** profile and the following manipulation is used to eliminate the propagation constant β:

$$\frac{1}{r}\frac{\partial}{\partial r}\left(r\frac{\partial e_{co}(r)}{\partial r}\right)+\left(k^2 n(r)^2-\beta^2+k^2{n_{co}}^2-k^2{n_{co}}^2-\frac{l^2}{r^2}\right)e_{co}(r)=0$$
$$\frac{1}{r}\frac{\partial}{\partial r}\left(r\frac{\partial e_{co}(r)}{\partial r}\right)+\left(k^2 n(r)^2-k^2{n_{co}}^2+\frac{V^2}{{a_{core}}^2}(1-b)-\frac{l^2}{r^2}\right)e_{co}(r)=0$$
$$\frac{1}{r}\frac{\partial}{\partial r}\left(r\frac{\partial e_{co}(r)}{\partial r}\right)+\left(k^2\left(n(r)^2-{n_{co}}^2\right)+\frac{V^2}{{a_{core}}^2}(1-b)-\frac{l^2}{r^2}\right)e_{co}(r)=0 \tag{36}$$

where $n(r)$ denotes the arbitrary profile of the **RI** in the core region of the fiber and n_{co} denotes the nominal **RI** of the core at the central axis of the fiber.

A similar manipulation is performed on the cladding differential equation knowing that the term $k^2n^2-\beta^2$ is defined as follows for the cladding region:

$$k^2{n_{cl}}^2-\beta^2=-\frac{V^2}{{a_{core}}^2}b$$

Similarly, Equation (35) is left in terms of $n(r)$ to accommodate an arbitrary cladding **RI** profile and the following manipulation is used to eliminate the propagation constant β:

$$\frac{1}{r}\frac{\partial}{\partial r}\left(r\frac{\partial e_{cl}(r)}{\partial r}\right)+\left(k^2 n(r)^2-\beta^2+k^2{n_{cl}}^2-k^2{n_{cl}}^2-\frac{l^2}{r^2}\right)e_{cl}(r)=0$$
$$\frac{1}{r}\frac{\partial}{\partial r}\left(r\frac{\partial e_{cl}(r)}{\partial r}\right)+\left(k^2 n(r)^2-k^2{n_{cl}}^2-\frac{V^2}{{a_{core}}^2}b-\frac{l^2}{r^2}\right)e_{cl}(r)=0$$
$$\frac{1}{r}\frac{\partial}{\partial r}\left(r\frac{\partial e_{cl}(r)}{\partial r}\right)+\left(k^2\left(n(r)^2-{n_{cl}}^2\right)-\frac{V^2}{{a_{core}}^2}b-\frac{l^2}{r^2}\right)e_{cl}(r)=0 \tag{37}$$

where $n(r)$ denotes the arbitrary profile of the **RI** in the cladding region of the fiber and n_{cl} denotes the nominal **RI** of the core at the central axis of the fiber.

Equations (36) and (37) represent the general differential equations governing the solution to the radial tangential field which defines the intensity distribution for any particular mode.

References

1. Iizuka, K. 11.2 THEORY OF STEP-INDEX FIBERS. In *ELEMENTS OF PHOTONICS, New Jersy, John Wiley & Sons*; 2002; pp. 132–162.
2. Chen, C.L. 2.3 Generalized Parameters. In *Foundation For Guided Wave Optics, Hoboken, New Jersey: John Wiley & Sons*; 2007; pp. 25–50.
